# Long-Term Fluoride Exchanges at Restoration Surfaces and Effects on Surface Mechanical Properties

**DOI:** 10.1155/2013/579039

**Published:** 2013-08-19

**Authors:** Steven Naoum, Elizabeth Martin, Ayman Ellakwa

**Affiliations:** ^1^Faculty of Dentistry, The University of Sydney, Australia; ^2^Westmead Oral Health Centre, Level 1, Faculty Office, Westmead Hospital, Darcy Road, Westmead, Sydney, NSW 2145, Australia; ^3^Faculty of Dentistry, Tanta University, Egypt

## Abstract

*Aim*. The aim of the study was to determine whether three fluoride containing resin composites could maintain fluoride release, fluoride recharge, and mechanical stability over long-term (18-month) aging. *Materials and Methods*. Fluoride containing composites Beautifil II, Gradia Direct X, Tetric EvoCeram, and glass ionomer Fuji IX Extra were analyzed. Specimens of each material were fabricated for two test groups: Group 1: bimonthly fluoride release/recharge analysis (*n* = 5); Group 2: hardness and elastic modulus analysis (*n* = 6). Nanoindentation was employed at 24 hours and at 1, 3, 6, 12, and 18 months. After 18 months, each specimen was immersed (recharged) in 5000 ppm NaF gel, and fluoride rerelease, hardness, and elastic modulus were measured. *Results.* Beautifil II and Gradia Direct X maintained fluoride release and recharge capability throughout 18-month aging (Beautifil II > Gradia Direct X > Tetric EvoCeram). The fluoride rerelease from Beautifil II following a 10-minute NaF recharge (at 18 months) was comparable to the long-term fluoride release from Fuji IX Extra. Elastic modulus and hardness did not change significantly (*P* > 0.05) with fluoride release, recharge, and water aging over 18 months for all three analyzed composites. *Conclusions.* The long-term fluoride release, fluoride recharge, and mechanical property stability of Beautifil II and Gradia Direct X render these composites suitable for load bearing restorations in high caries risk patients. * Clinical Relevance.* The ability for Beautifil II and Gradia Direct X to maintain fluoride release and fluoride recharge capability, despite long-term aging, raises the potential for unrestored tooth surfaces in contact with Beautifil II or Gradia Direct X restorations to demonstrate a reduced rate of caries incidence compared to unrestored surfaces adjacent to conventional nonfluoride containing composites.

## 1. Introduction 

It is well established that topically applied fluoride ions, through integration into the mineral component of enamel and dentin, can function to reduce the incidence and progression of dental caries [1, 2]. Fluoride complexes have the ability to promote dental tissue remineralization [3, 4] in addition to increasing the resistance of tooth structure to demineralization [5]. Fluoride can be made available to tooth surfaces through several methods including via dentifrices, mouth rinses, and fluoridated water intake. Additionally, fluoride can become available to a tooth surface via fluoride release from a restorative material in close proximity. Notably, several *in vivo* studies have concluded that the fluoride release from restorative materials is able to reduce the incidence of caries affecting unrestored tooth surfaces [6–8]. In particular, long-term clinical studies have demonstrated that unrestored proximal surfaces contacting fluoride releasing class II restorations can exhibit a lower incidence of caries compared to surfaces contacting non-fluoridated restorations [6–8]. This suggests that employing restorative materials capable of fluoride release can be especially advantageous in the treatment of high caries risk patients. 

The possibility of restorative material fluoride release facilitating a reduction in caries incidence has long been a heralded advantage of glass ionomer restorative materials [9, 10] and has resulted in several material classes being developed which combine glass ionomers and resin matrices. The most recent attempt to integrate the components of glass ionomers within a resin matrix is the giomer material class. Giomers are resin composites which contain prereacted glass (PRG) ionomer filler particles within a resin matrix [11, 12]. PRG filler particles are formed by an acid-base reaction between fluoro-boro-alumino silicate glass particles and polyalkenoic acid in the presence of water. This process produces fluoridated glass particles surrounded by a glass ionomer hydrogel. PRG particles are integrated into a resin matrix following silane treatment in the same manner as conventional composites [12, 13]. Prereacted glass ionomer particles thus provide giomers with the potential to exhibit physical and aesthetic properties comparable to conventional composites and simultaneously provide tooth structure in close proximity with fluoride complexes that can promote tooth remineralization. Significantly, while the fluoride release and fluoride recharge of giomer restorative materials has been demonstrated over a short period [14], the capacity of giomers to demonstrate sustained fluoride release and fluoride recharge capability over long-term aging has not been assessed. Notably the ability of a restorative material to sustain fluoride release and fluoride recharge despite long-term aging has been suggested as essential if a restorative material's fluoride release is to contribute to a clinically identifiable reduction in caries incidence [9, 15]. Additionally, no study has been undertaken to assess if the processes of fluoride release and fluoride recharge affect the mechanical stability giomers long-term.

The aim of the present *in vitro* study was to determine whether three fluoride containing resin composites including one of the giomer classification could maintain fluoride release, fluoride recharge, and mechanical stability over long-term (18-month) aging. The null hypothesis was that the three fluoride containing composites would not maintain fluoride release, fluoride recharge, and mechanical stability over 18 month aging. The present study also sought to identify whether the fluoride rerelease following recharge after long-term aging demonstrated by the assessed composites was comparable to the long-term intrinsic fluoride release from glass ionomers, as it is the long-term intrinsic fluoride release from glass ionomers that any caries inhibitive activity of glass ionomers can be attributed [9, 14, 15]. 

## 2. Methods

Three fluoride containing resin composites were analyzed in this study: giomer Beautifil II (Shofu Inc, Kyoto, Japan; Lot 060854; A2), Tetric EvoCeram (Ivoclar Vivadent, Schaan, Liechtenstein; Lot L24180; A2), and Gradia Direct X (GC Co., Tokyo, Japan; Lot 0805142; A3). Fuji IX Extra (GC Co., Tokyo, Japan; Lot 0804151; A3) was also analyzed for comparison (Table 1). Two specimen groups of each material were fabricated for analysis (Table 2). 

### 2.1. Group 1: Fluoride Release and Recharge Analysis

Five disc-shaped specimens of each material (inner diameter 10.0 mm, depth 1.5 mm) were prepared for fluoride release and fluoride recharge measurements using a polytetrafluoroethylene mold. Following material dispensing, a glass plate (thickness 1.0 mm) was placed over the material, and finger pressure was applied to ensure removal of air and material excess. Curing of each composite specimen was completed using a halogen curing light (Optilux 501, Kerr Co., Orange, USA) at a measured intensity of 400 mW/cm^2^ (Curing Radiometer, Demetron Research Corporation, Danbury, USA) for 40 seconds. Glass ionomer specimens were retained in the mold for 10 minutes after mixing. All specimens were kept at 100% relative humidity for 30 minutes at 37°C following fabrication before light polishing of specimen edges with dry 600 grit silicon carbide paper. The dimensions of each specimen were measured before placement into the storage media. 

Group 1 specimens were aged in individual plastic jars containing 20 mL of deionized water (Milli-Q plus, 18.2 Mcm, Millipore, NY, USA) for 18 months at 37°C. The fluoride ion release from each specimen was measured bimonthly. Following each measurement, the storage medium for each specimen was discarded, and specimens were placed in a clean jar containing 20 mL of deionized water. After 18-month aging, each specimen was immersed (recharged) in 5000 ppm neutral sodium fluoride gel (NeutraFluor 5000 Plus, Colgate, NY, USA) for 10 minutes. Following recharge, each specimen was thoroughly rinsed using deionized water to remove any adsorbed material before being placed in new aging solution. The fluoride rerelease from each specimen was measured at two months after this single recharge episode.

To measure specimen fluoride ion release (and rerelease after recharge) total ionic strength adjustment buffer II solution was added to each specimen's storage solution, following specimen removal. A fluoride ion selective electrode (Radiometer Analytical, Copenhagen, Denmark) was used to measure the fluoride concentration of the aged solutions. Standards containing 0.025–0.25 mg/L fluoride in 0.025 mg/L fluoride steps were used for calibration at each testing interval. The results attained were expressed as the quantity of fluoride released per unit area of specimen (*μ*g/cm^2^). 

### 2.2. Group 2: Mechanical Properties Analysis

A method similar to that used by Naoum et al. [14] was employed to measure the elastic modulus and hardness of the analyzed materials. Six specimens of each material were fabricated for mechanical properties analysis, forming Group 2 specimens. Group 2 specimens were prepared in an identical fashion to Group 1 specimens, except that a mold of dimensions 7.0 mm × 2.0 mm was used for logistical reasons. 

Once fabricated, each specimen was placed in 20 mL of storage media and aged for 18 months at 37°C; 3 specimens were stored in deionized water, and 3 specimens were stored in lactic acid (pH 4.0). Specimens were stored in lactic acid in addition to deionized water so that any effect of filler particle dissolution upon material mechanical properties could be realized over the analysis period of the study. The aging solutions were renewed monthly to ensure that specimens were exposed to a pH as constant as possible over the 18-month. Following 18 months aging, each specimen was immersed (recharged) in 5000 ppm neutral sodium fluoride gel (NeutraFluor 5000 Plus, Colgate, USA) for 1 hour; an immersion time longer than used for Group 1 to maximize any effect of the recharge process within the limitations of the present study. Following recharge, each specimen was thoroughly rinsed using deionized water to remove any adsorbed material before being returned to its storage solution.

The hardness and elastic modulus of each specimen were measured via nanoindentation at 24 hours, 1 month, 3 months, 6 months, 12 months, and 18 months after fabrication. At twenty-four hours after the 18-month fluoride recharge episode, the hardness and elastic modulus of each specimen were again measured.

Indentations were made using an ultramicroindentation system (UMIS 2000, CSIRO, Canberra, Australia). A calibrated diamond Berkovich indenter tip was used to apply loads of 50 mN to the specimen surface, 25 *μ*m apart. In order to minimize creep during unloading and produce more reliable elastic modulus values, the maximum force for each indent was held on the surface for 30 seconds before load, and depth readings were made [16]. Each specimen was exposed to 16 indents to provide 48 data points for each material in each storage medium at each testing time. The hardness and elastic modulus for each material were calculated using the software associated with the UMIS. The hardness was calculated by dividing the applied load by the surface area. The elastic modulus was calculated by [16]

(1) $$\begin{array}{c} \frac{1}{E_{r}}=\frac{1-v_{m}^{2}}{E_{m}}+\frac{1-v_{i}^{2}}{E_{i}}, \end{array}$$

where *E*_*r*_ is the reduced modulus from the nanoindenter; determined from the recovery rate on unloading at maximum load, *v*_*m*_ and *E*_*m*_ are Poisson's ratio and elastic modulus of the composite material; *V*_*i*_ and *E*_*i*_ are the elastic modulus and Poisson's ratio of the indenter. Poisson's ratio for each material was adapted from findings by Chung et al. [17]. 

### 2.3. Statistical Analysis

Two-way factorial analysis of variance (ANOVA) and posthoc (Tukey) testing were used to assess the influence of storage media (2 levels) and material type (4 levels) on the hardness and elastic modulus of the assessed materials. One-way factorial analysis of variance (ANOVA) was used to assess the influence of material type (4 levels) on fluoride release and fluoride recharge. The level of significance was set at *P* = 0.05. 

## 3. Results

**Figure 1:**
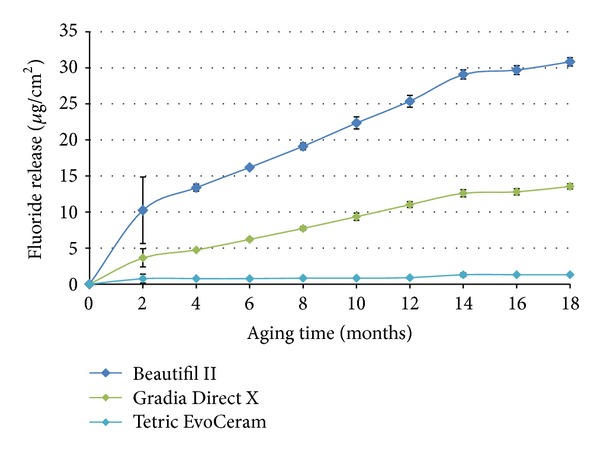
The cumulative fluoride release by each composite over 18 months of aging.

**Figure 2:**
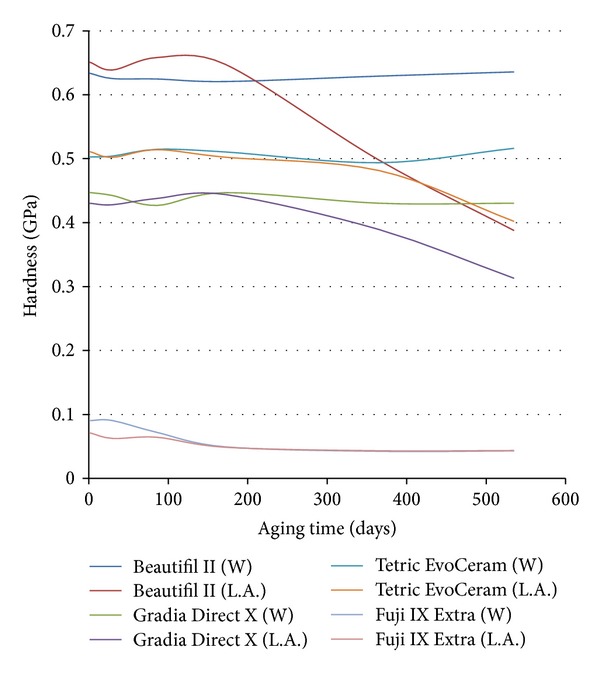
The hardness of each material over 18-month aging in lactic acid ph 4 (L.A.) and deionized water (W).

**Figure 3:**
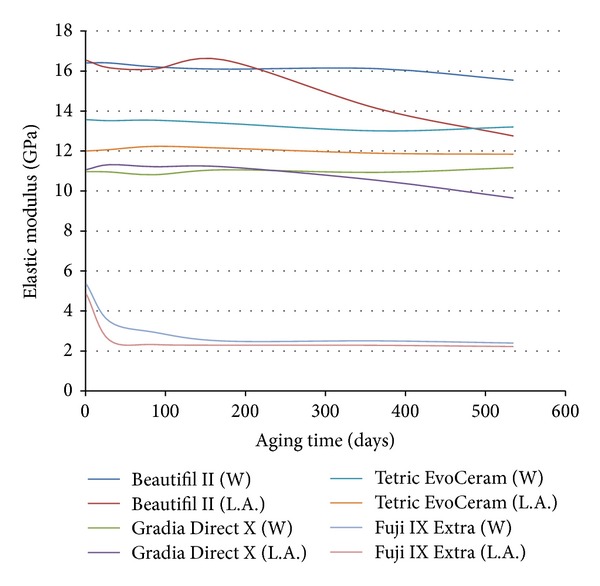
The elastic modulus of each material over 18-month aging in lactic acid ph 4 (L.A.) and deionized water (W).

**Figure 4:**
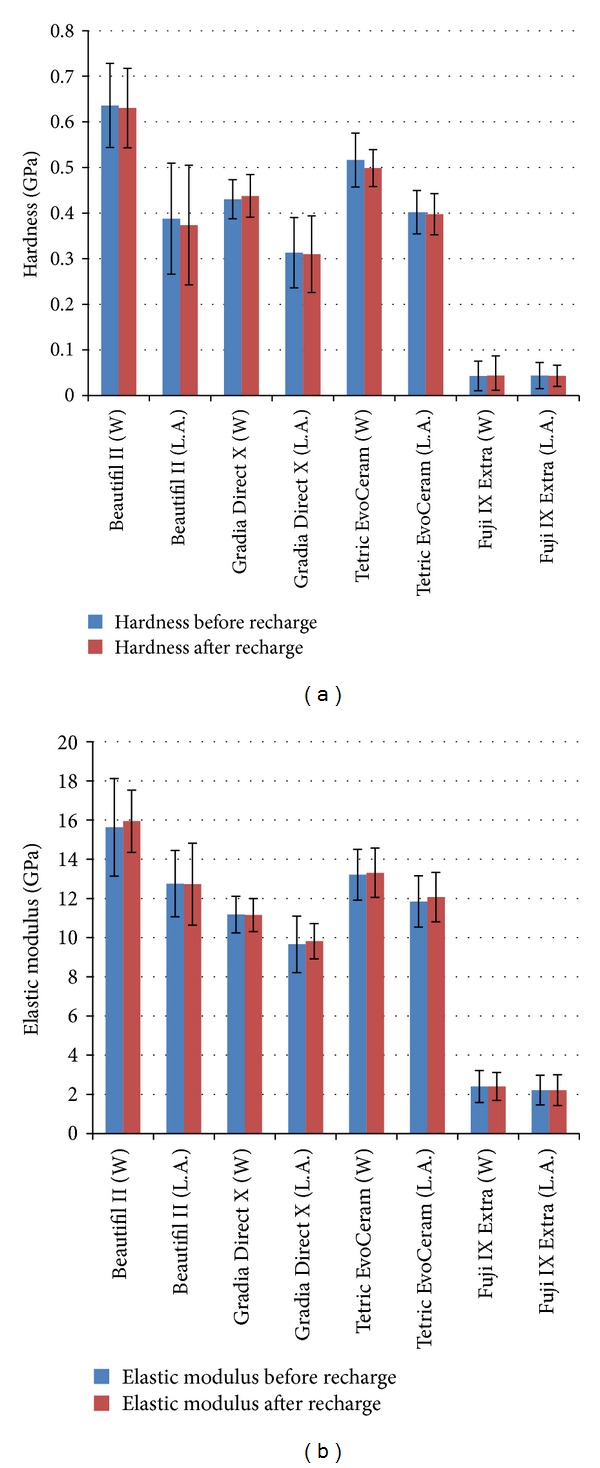
(a) The effect of 1-hour fluoride recharge on composite hardness after 18 months of aging in lactic acid ph 4 (L.A.) and deionized water (W). (b) The effect of 1-hour fluoride recharge on composite elastic modulus after 18 months of aging in lactic acid ph 4 (L.A.) and deionized water (W).

The results from the present study are displayed in Figures 1–4 and Tables 3 and 4.  Figure 1 shows the cumulative fluoride release exhibited by each composite over 18-month aging. Beautifil II and Gradia Direct X demonstrated sustained fluoride ion release for the entire 18 months of analysis. The cumulative fluoride ion release by Beautifil II at the completion of the 18-month aging was significantly (*P* < 0.05) greater than the release by Gradia Direct X and Tetric EvoCeram (Gradia Direct X > Tetric EvoCeram). Tetric EvoCeram did not exhibit fluoride ion release after 14-month aging. All three materials released the greatest quantity of fluoride ions during the first two months of aging.

Table 3 depicts the fluoride ion rerelease by each composite after fluoride recharge (10 minutes, 5000 ppm NaF) at 18 months aging. All three composites demonstrated fluoride recharge capability after 18 months aging; all three composites rerelease fluoride ions following fluoride application (recharge). Beautifil II exhibited a significantly (*P* < 0.05) greater fluoride ion rerelease following the recharge treatment at 18 months of aging compared to both Gradia Direct X and Tetric EvoCeram (Gradia Direct X >Tetric EvoCeram).

Table 4 shows the intrinsic bimonthly fluoride ion release by Fuji IX Extra over the 18 months of the study. The intrinsic fluoride release by Fuji IX Extra was significantly (*P* < 0.05) greater than that of the three analyzed composites. The fluoride rereleased from Beautifil II in the 2 months following fluoride recharge at 18 months of aging was comparable to the intrinsic fluoride released by Fuji IX Extra during 15-16 and 17-18 months. 

Figures 2 and 3 depict the elastic modulus and hardness of each material aged in deionized water and lactic acid over 18-month aging. The elastic modulus and hardness of the three composites did not change significantly (*P* > 0.05) with fluoride release, water storage, or water uptake over the 18-month analysis period. However, lactic acid storage significantly (*P* < 0.05) reduced the hardness of all three composites after 6-month aging and caused a reduction in the elastic modulus of Beautifil II and Gradia Direct X after 6-month aging. Exposure to a 1-hour episode of fluoride recharge after 18-month aging did not significantly (*P* > 0.05) affect the hardness or elastic modulus of the tested materials (Figure 4). The hardness and elastic modulus of Fuji IX Extra were significantly (*P* > 0.05) lower than the analyzed composites at each testing interval (Figures 2–4). 

The results of the present study indicate that the null hypothesis was partially accepted: Beautifil II and Gradia Direct X maintained fluoride release and fluoride recharge capability for the 18 months of the study; all three assessed composites maintained mechanical property stability in deionized water over 18-month aging.

## 4. Discussion 

The difficulty and reluctance of patients to undertake preventive measures to ensure that interproximal tooth surfaces remain bacteria free cause interproximal tooth sites to be susceptible to caries incidence [18–20]. This difficulty can be further exacerbated when a proximal tooth surface is restored directly using a resin composite material; composite polymerization contraction can impede the recreation of self cleansing interproximal contacts [21]. Significantly, it has been shown that unrestored proximal tooth surfaces contacting fluoride releasing class II restorations can exhibit a lower incidence of new caries in comparison to unrestored surfaces contacting nonfluoride containing restorative materials [6–8]. This differential is attributed to the ability of a fluoride containing restorative material to sustain fluoride release over time rather than an ability to demonstrate a high “burst” of fluoride release immediately following placement [9, 15, 22]. Such is a consequence of the nature of the carious process; carious tooth destruction develops as demineralization, exceeds remineralization over months to years rather than at a single point in time [4]. The results of the present study indicate that Beautifil II and Gradia Direct X have this long-term sustained fluoride releasing capability.

The observed significantly (*P* < 0.05) greater fluoride release demonstrated by giomer Beautifil II in comparison to Gradia Direct X and Tetric EvoCeram can be attributed to the fluoride releasing ability of PRG filler particles; all three materials have comparable filler loading and resin matrix hydrophobicity. While Gradia Direct X, Tetric EvoCeram, and Beautifil II have the ability to release fluoride into their resin matrix and surrounding media following filler particle surface dissolution [23], Beautifil II has an additional source of fluoride for release; the fluoride complexes within their glass ionomer hydrogel of PRG particles [24]. Further, the acidified water within the hydrogel surrounding the inner glass of PRG particles facilitates Beautifil II fluoride release through additional dissolution of the fluoride containing glass core [14, 15, 25].

The PRG particles within Beautifil II are also responsible for the significantly (*P* < 0.05) greater fluoride recharge demonstrated by giomer Beautifil II in comparison to Gradia Direct X and Tetric EvoCeram. The ability of a material to exhibit fluoride recharge depends on its ability to retain fluoride [14, 26, 27]. The relatively hydrophobic nature of the resin matrices of all three analyzed composites implicates the glass ionomer hydrogel of PRG particles as the key reason for the additional recharge demonstrated by Beautifil II compared to Tetric EvoCeram and Gradia Direct X. The hydrogel of PRG particles exhibits a higher permeability and porosity than resin matrices [14, 27, 28]. Consequently, this hydrogel provides Beautifil II with areas within its structure capable of greater fluoride uptake relative to a composite not containing a glass ionomer phase [14]. 

The potential clinical significance of the sustained long-term fluoride recharge and rerelease capability of Beautifil II is brought into view when considering it in the context of the observed intrinsic fluoride release produced by glass ionomer Fuji IX Extra. In the present study, following only a single episode of fluoride recharge after 18-month aging (5000 ppm NaF for 10 minutes), the concentration of fluoride ions re-released by Beautifil II in the subsequent two months was comparable to the intrinsic bimonthly release by Fuji IX Extra between 14–18 months. Therefore, since it is the long term intrinsic “plateau” release of glass ionomers that is responsible for any caries inhibitive activity of glass ionomers [29], the present study indicates that, should a regular fluoride recharge regime be implemented by patients, Beautifil II has the potential to exhibit a fluoride rerelease comparable to the long-term “plateau” release of glass ionomers and a potentially comparable caries inhibitive activity that such release can generate [6–8, 29].

The possibility of this favourable clinical outcome is also supported by a recently completed assessment analyzing the same three composites as evaluated in the present study [14]. Naoum et al. [14] observed that, during the 24 hours following a 5-minute application of 5000 ppm NaF gel, Beautifil II re-released fluoride ions at a rate of 3.7 *μ*g/cm^2^ per day. Notably this rerelease rate was able to be repeated following 3 consecutive recharge episodes that were separated by a week interval, demonstrating maintenance of rechargeability with consecutive recharge episodes. Therefore should a daily 5 minute fluoride recharge application of 5000 ppm NaF be employed by patients from the time of restoration placement, a measure feasibly instituted as part of an individual's routine oral hygiene, the fluoride rerelease from Beautifil II could approach the intrinsic “plateau” release from glass ionomers within one month of tooth restoration [14]. Importantly, the present study provides clinicians with confidence that the fluoride rerelease following recharge from Beautifil II can continue as the restoration ages so enabling these rerelease levels to be sustained over time.

The mechanical properties (elastic modulus and hardness) of all three resin composites maintained stability over 18-month aging in water. This stability indicates that, under oral conditions when salivary pH is above that required to initiate caries, little degradation of the constituents of the assessed resin composites will occur as a result of salivary pH alone. However, all three composites exhibited a significant (*P* < 0.05) reduction in hardness values after 6 months of lactic acid storage, with Beautifil II and Gradia Direct X also demonstrating a decline in elastic modulus after 6 months of acid aging. Fluid of low pH absorbed by resin composites can result in resin matrix degradation, filler particle degradation, and hydrolysis of the Si–O bonds that link the filler and resin matrix [30]. It is likely that all three processes affected the constituents of the three analysed composites [30]. 

Clinically this reduction in mechanical properties under acidic conditions can have implications for practitioners when treating particular cohorts of medically compromised patients. When treating patients suffering from chronic hypoxia or exocrine conditions that can lead to a sustained reduction in salivary pH, it is likely that the composites in the present study are susceptible to long-term physical degradation with aging. Consequently, when using the analysed composites in clinical practise, salivary analysis along with measures to elevate salivary pH should accompany a prescription of regular fluoride recharge. 

In contrast to the mechanical property stability of the three composites aged in water in the present study, the hardness and elastic modulus of glass ionomer Fuji IX Extra degraded with time. The observed mechanical property degradation of Fuji IX Extra in both neutral and acidic conditions can be attributed to the permeability and porosity of glass ionomers [31]. Being permeable, glass ionomers readily uptake storage media [32]. While this enhances fluoride release and fluoride recharge [32, 33], it can also cause breakdown of the nonsilanized glass fillers within glass ionomers and produce a reduction in mechanical properties. This degradation, as well as the absolute values of glass ionomer physical properties, limits the use of glass ionomers in load bearing restorations.

In closing it is appropriate to acknowledge the limitations of the present study. Firstly to gain a more thorough assessment of the effect of fluoride recharge on mechanical property stability, an investigation of the impact of repeated fluoride recharge on material mechanical properties is required. The action of sodium fluoride gel upon glass filler particles can cause glass dissolution as well as disintegration of the matrix around composite filler particles [34, 35]. Consequently, despite the observed mechanical property stability of the assessed composites following a recharge episode of duration far longer than would be prescribed to patients (1 hour), an investigation to ascertain the frequency and concentration of fluoride recharge that initiates composite physical property degradation is planned. Secondly, with regard to the fluoride release and fluoride recharge analysis of the present study the time span between testing points presents a limitation. While the decision not to undertake daily analysis of fluoride release behavior was made due to a recent study assessing the daily fluoride release from the composites in the present study [14], daily release analysis would be a helpful adjunct to the presented results. 

However despite these acknowledged limitations, with long-term clinical trials now indicating that fluoride released from placed restorations can reduce caries incidence affecting contacting tooth surfaces [6–8], the findings of the present study do provide clinicians with helpful information; in the context of a regularly applied fluoride recharge regime, the fluoride release and rerelease from Beautifil II (and possibly Gradia Direct X) has the potential to reduce new caries incidence at unrestored contacting surfaces. 

## 5. Conclusion

Within the limitations of the present *in vitro* study it can be concluded that resin composites Beautifil II and Gradia Direct X have the ability to sustain intrinsic fluoride release and maintain fluoride recharge capability despite long-term aging. In the context that a patient regularly applies fluoride to placed Beautifil II restorations, Beautifil II is capable of rereleasing fluoride at a rate comparable to the long-term fluoride release from Fuji IX Extra. Beautifil II, Gradia Direct X, and Tetric EvoCeram are capable of maintaining mechanical property stability despite long-term water aging, fluoride release and fluoride recharge.

## 6. Clinical Significance

The ability for Beautifil II and Gradia Direct X to sustain intrinsic fluoride release and maintain fluoride recharge capability despite long-term aging raises the potential for unrestored tooth surfaces in contact with Beautifil II (and possibly Gradia Direct X) restorations to demonstrate a reduced rate of caries incidence compared to surfaces adjacent to conventional nonfluoride containing composites. The exhibited mechanical property stability of Beautifil II and Gradia Direct X, despite long-term water aging, fluoride release, and fluoride recharge, indicates that Beautifil II and Gradia Direct X are suitable for load bearing restorations in “high caries risk” patients, where fluoride release is advantageous and placement of glass ionomers is contraindicated. 

## Figures and Tables

**Table 1:** Description of analyzed materials.

Material	Key contents	Manufacturer
Tetric EvoCeram Lot L24180	Filler particles consisting of barium glass, ytterbium trifluoride, mixed oxide and prepolymer, and unspecified dimethacrylate monomers (17 wt%)	Ivoclar Vivadent, Schaan, Liechtenstein
Gradia Direct X Lot 0805142	Fluoroaluminosilicate glass, prepolymerised filler, silica, UDMA, and unspecified dimethacrylate comonomers (23 wt%).	GC Co., Tokyo, Japan
Beautifil II Lot 060854	S-PRG glass filler, fluoride containing fluoro-boro-alumino silicate glass filler particles, TEGDMA, and Bis-GMA (17 wt%)	Shofu Inc., Kyoto, Japan
Fuji IX Extra Lot 0804151	Fluoroaluminosilicate glass, copolymer of acrylic and maleic acid, tartaric acid, and water	GC Co., Tokyo, Japan

S-PRG filler: surface reaction-type prereacted glass ionomer; Bis-GMA: 2,2-bis [4-(2′-hydroxy-3′-methacryloxypropoxy)phenyl]propane; TEGDMA: triethylene glycol dimethacrylate; UDMA: urethane dimethacrylate.

**Table 2:** Method overview.

Analysis undertaken	Group 1 (fluoride release/recharge)	Group 2 (mechanical properties; elastic modulus/hardness)
Specimen number per material	3	6
Storage media	Deionized water	Deionized water (3) Lactic acid pH 4 (3)
Analysis times over 18 mth aging	Bimonthly (0, 2, 4, 6, 8, 10, 12, 14, 16, and 18 mths)	24 hrs, 1 mth, 3 mths, 6 mths, 12 mths, and 18 mths
Time elapsed following 18 mth F^−^ recharge before recharge analysis	2 months	24 hours

**Table 3:** The fluoride ion rerelease by each composite after fluoride recharge (10 minutes, 5000 ppm) at 18 months of aging.

Composite material	Fluoride ion rerelease following recharge (*μ*g/cm^2^)
Beautifil II	31.7
Gradia Direct X	11.1
Tetric EvoCeram	6.0

**Table 4:** The intrinsic bimonthly fluoride ion release by Fuji IX Extra over 18 months of aging.

Aging time (months)	0–2	3-4	5-6	7-8	9-10	11-12	13-14	15-16	17-18
Fluoride ion release (*μ*g/cm^2^)	145.7	102.9	80.1	61.2	56.1	50.0	48.0	40.1	38.5

## References

[1] Ten Cate JM (1999). Current concepts on the theories of the mechanism of action of fluoride. *Acta Odontologica Scandinavica*.

[2] González-Cabezas C (2010). The chemistry of caries: remineralization and demineralization events with direct clinical relevance. *Dental Clinics of North America*.

[3] Fejerskov O, Clarkson BH, Fejerskov O, Ekstrand J, Burt BA (1996). Dynamics of caries lesion formation. *Fluoride in Dentistry*.

[4] Kidd E, Joyston-Bechal S (2005). *Aetiology of Dental Caries Essentials of Dental Caries. Disease and Its Management*.

[5] Evans W (1989). Conference report: a joint IADR/ORCA international symposium- fluorides: mechanisms of action and recommendations for use. *Journal of Dental Research*.

[6] Qvist V, Poulsen A, Teglers PT, Mjör IA (2010). Fluorides leaching from restorative materials and the effect on adjacent teeth. *International Dental Journal*.

[7] Qvist V, Laurberg L, Poulsen A, Teglers PT (2004). Class II restorations in primary teeth: 7-year study on three resin-modified glass ionomer cements and a compomer. *European Journal of Oral Sciences*.

[8] Qvist V, Manscher E, Teglers PT (2004). Resin-modified and conventional glass ionomer restorations in primary teeth: 8-Year results. *Journal of Dentistry*.

[9] Wiegand A, Buchalla W, Attin T (2007). Review on fluoride-releasing restorative materials—fluoride release and uptake characteristics, antibacterial activity and influence on caries formation. *Dental Materials*.

[10] Tyas MJ (1992). Clinical studies related to glass ionomers. *Operative Dentistry*.

[11] Tay FR, Pashley EL, Huang C (2001). The glass-ionomer phase in resin-based restorative materials. *Journal of Dental Research*.

[12] Roberts TA, Miyai K, Ikemura K, Fuchigami K, Kitamura T (1999). Fluoride ion sustained release preformed glass ionomer filler and dental composite containing the same name.

[13] Gordan VV, Mondragon E, Watson RE, Garvan C, Mjör IA (2007). A clinical evaluation of a self-etching primer and a giomer restorative material: results at eight years. *Journal of the American Dental Association*.

[14] Naoum S, Ellakwa A, Martin F, Swain M (2011). Fluoride release, recharge and mechanical property stability of various fluoride-containing resin composites. *Operative Dentistry*.

[15] Attar N, Turgut MD (2003). Fluoride release and uptake capacities of fluoride-releasing restorative materials. *Operative Dentistry*.

[16] Angker L, Nockolds C, Swain MV, Kilpatrick N (2004). Correlating the mechanical properties to the mineral content of carious dentine—a comparative study using an ultra-micro indentation system (UMIS) and SEM-BSE signals. *Archives of Oral Biology*.

[17] Chung SM, Yap AUJ, Koh WK, Tsai KT, Lim CT (2004). Measurement of Poisson’s ratio of dental composite restorative materials. *Biomaterials*.

[18] Mejàre I, Källestål C, Stenlund H, Johansson H (1998). Caries development from 11 to 22 years of age: a prospective radiographic study. Prevalence and distribution. *Caries Research*.

[19] Mejàre I, Stenlund H (2000). Caries rates for the mesial surface of the first permanent molar and the distal surface of the second primary molar from 6 to 12 years of age in Sweden. *Caries Research*.

[20] Hintze H (1997). Caries behaviour in Danish teenagers: a longitudinal radiographic study. *International Journal of Paediatric Dentistry*.

[21] El-Badrawy WA, Leung BW, El-Mowafy O, Rubo JH, Rubo MH (2003). Evaluation of proximal contacts of posterior composite restorations with 4 placement techniques. *Journal Canadian Dental Association*.

[22] Toumba KJ, Curzon ME (1993). Slow-release fluoride. *Caries Research*.

[23] Itota T, Carrick TE, Yoshiyama M, McCabe JF (2004). Fluoride release and recharge in giomer, compomer and resin composite. *Dental Materials*.

[24] Tay FR, Pashley EL, Huang C (2001). The glass-ionomer phase in resin-based restorative materials. *Journal of Dental Research*.

[25] De Moor RJ, Verbeeck RM, De Maeyer EA (1996). Fluoride release profiles of restorative glass ionomer formulations. *Dental Materials*.

[26] Han L, Cv E, Li M (2002). Effect of fluoride mouth rinse on fluoride releasing and recharging from aesthetic dental materials. *Dental Materials Journal*.

[27] Attin T, Buchalla W, Siewert C, Hellwig E (1999). Fluoride release/uptake of polyacid-modified resin composites (compomers) in neutral and acidic buffer solutions. *Journal of Oral Rehabilitation*.

[28] Preston AJ, Agalamanyi EA, Higham SM, Mair LH (2003). The recharge of esthetic dental restorative materials with fluoride in vitro—two years’ results. *Dental Materials*.

[29] Williams JA, Billington RW, Pearson GJ (2001). A long term study of fluoride release from metal-containing conventional and resin-modified glass-ionomer cements. *Journal of Oral Rehabilitation*.

[30] Drummond JL, Andronova K, Al-Turki LI, Slaughter LD (2004). Leaching and mechanical properties characterization of dental composites. *Journal of Biomedical Materials Research B*.

[31] Nicholson JW, Czarnecka B (2004). The release of ions by compomers under neutral and acidic conditions. *Journal of Oral Rehabilitation*.

[32] Preston AJ, Higham SM, Agalamanyi EA, Mair LH (1999). Fluoride recharge of aesthetic dental materials. *Journal of Oral Rehabilitation*.

[33] Xu X, Burgess JO (2003). Compressive strength, fluoride release and recharge of fluoride-releasing materials. *Biomaterials*.

[34] De Witte AMJC, De Maeyer EAP, Verbeeck RMH (2003). Surface roughening of glass ionomer cements by neutral NaF solutions. *Biomaterials*.

[35] Hadley PC, Billington RW, Pearson GJ, Williams JA (2000). Effect of monovalent ions in glass ionomer cements on their interaction with sodium fluoride solution. *Biomaterials*.

